# Differential gene expression profiles between two subtypes of ischemic stroke with blood stasis syndromes

**DOI:** 10.18632/oncotarget.22877

**Published:** 2017-12-04

**Authors:** Tian-Long Liu, Min-Na Liu, Xin-Liang Xu, Wen-Xing Liu, Pei-Jin Shang, Xiao-Hu Zhai, Hang Xu, Yi Ding, Yu-Wen Li, Ai-Dong Wen

**Affiliations:** ^1^ Department of Pharmacy, Xijing Hospital, Fourth Military Medical University, Xi’an, China; ^2^ Department of Pharmacy, 25th Hospital of PLA, Jiuquan, China; ^3^ Department of Nephrology, Xijing Hospital, Fourth Military Medical University, Xi’an, China; ^4^ State Key Laboratory of Cancer Biology, Fourth Military Medical University, Xi’an, China; ^5^ School of Medicine and Life Sciences, University of Jinan-Shandong Academy of Medical Sciences, Jinan, China; ^6^ Department of Traumatic Surgery, Jining No.1 Peoples Hospital, Jining, China; ^7^ Department of Pharmacy, The First Affiliated Hospital of SooChow University, Suzhou, China

**Keywords:** ischemic stroke, blood stasis syndrome, traditional Chinese medicine, transcriptomics, network analysis

## Abstract

Ischemic stroke is a cerebrovascular thrombotic disease with high morbidity and mortality. Qi deficiency blood stasis (QDBS) and Yin deficiency blood stasis (YDBS) are the two major subtypes of ischemic stroke according to the theories of traditional Chinese medicine. This study was conducted to distinguish these two syndromes at transcriptomics level and explore the underlying mechanisms. Male rats were randomly divided into three groups: sham group, QDBS/MCAO group and YDBS/MCAO group. Morphological changes were assessed after 24 h of reperfusion. Microarray analysis with circulating mRNA was then performed to identify differential gene expression profile, gene ontology and pathway enrichment analyses were carried out to predict the gene function, gene co-expression and pathway networks were constructed to identify the hub biomarkers, which were further validated by western blotting and Tunel staining analysis. Three subsets of dysregulated genes were acquired, including 445 QDBS-specific genes, 490 YDBS-specific genes and 1676 blood stasis common genes. Our work reveals for the first time that T cell receptor, MAPK and apoptosis pathway were identified as the hub pathways based on the pathway networks, while *Nfκb1*, *Egfr* and *Casp3* were recognized as the hub genes by co-expression networks. This research helps contribute to a clearer understanding of the pathological characteristics of ischemic stroke with QDBS and YDBS syndrome, the proposed biomarkers might provide insight into the accurate diagnose and proper treatment for ischemic stroke with blood stasis syndrome.

## INTRODUCTION

Ischemic stroke is a serious and life-threatening cerebrovascular thrombotic disease [1]. On average, someone has a stroke every 40 seconds and someone dies of stroke approximately every 4 minutes [2]. Unfortunately, despite of the use of aspirin and thrombolytics, effect is limited due to its extremely narrow therapeutic time window [3]. Traditional Chinese Medicine (TCM) might help to overcome ischemic stroke since its acknowledged efficacy [4]. Based on TCM theory, blood stasis syndrome (BSS) is the major phenotype in ischemic stroke [5], it means the circulation of blood is not smooth or blood flow is stagnant and forms stasis [6]. Modern pathology shows that blood stasis is generally manifested by cardio-cerebrovascular diseases such as myocardial infraction and local ischemia [7].

Ischemic stroke includes several subtypes, in which Qi deficiency blood stasis (QDBS) and Yin deficiency blood stasis (YDBS) are two of the most common syndromes [8]. The criterion of diagnostic and evaluation of QDBS and YDBS is based on the “Ischemic Stroke TCM Syndrome Factor Diagnostic Scale” and “Ischemic Stroke TCM Syndrome Factor Evaluation Scale” [9, 10]. The pathogenic mechanism between QDBS and YDBS is quite different [11]. According to TCM theory, “Qi” refers to the vital energy which flows within the body, it maintains blood circulation, digesting food, warms the body, as well as fights against diseases. Patients with “Qi-deficiency” are characterized by short breath, dizziness, spiritlessness, hypodynamia, light colored tongue and thread pulse [12]. While “Yin” refers to blood, other bodily fluids and ‘‘essence’’. Patients with “Yin-deficiency” usually manifests as rapid-small pulse, sleeplessness with irritability, red tongue with reduced or no coating, dry mouth, facial flushing and night sweats, etc. [13]. Syndrome differentiation, a traditional diagnostic method to categorize patients’ syndromes based on their physiological and clinical characteristics, is the foundation of personalized treatment in clinical practice [14]. However, since syndrome identification is mainly based on the experience of practitioners instead of a biomedical disease diagnosis, which has the drawbacks of subjectivity and variability [15]. Thus an accurate estimation with objective indexes for syndrome differentiation is crucial.

Advanced omics technology is considered as a holistic and efficient tool to study the syndrome of TCM, it can be used as a bridge between TCM and western medicine [16]. Dai et al. first proposed a new macro-micro concept, namely, “Syndrome-Omics”, which was defined as “a systematic approach for targeting individual patient, guiding treatment, and predicting the outcome of personalized treatment by global NET-Markers on the basis of syndrome identification and treatment” [17]. Subnetwork markers have proven to be more robust and reliable than individual marker genes selected without network information, and achieve higher accuracy in disease classification [18]. The combinations of syndrome and omics have been extensively used to build up molecular networks of TCM syndrome [19, 20], which would help to decipher the mechanism of TCM syndrome differentiation and find the personalized medicine for individual patient.

The syndrome differentiation has close intrinsic relations with the difference of gene expression which is the research missions of transcriptomics [21]. Numerous researches on circulating mRNA have been performed to identify diagnostic biomarkers with different syndromes [22, 23]. The development of diseases in hemorheology, platelet function and vascular endothelium injury constitute the pathological basis for cerebral vascular diseases [24]. Thus the expression profile in circulating could reflect the essence of BSS, and the mRNA in circulating could be potential biomarkers of BSS. In order to explore the mechanism of BSS, rat ischemic stroke model with BSS has been established in our laboratory [25]. However, few previous studies have emphasized the value of circulating mRNA as potential biomarkers for making a distinction between QDBS and YDBS of ischemic stroke.

**Figure 1:**
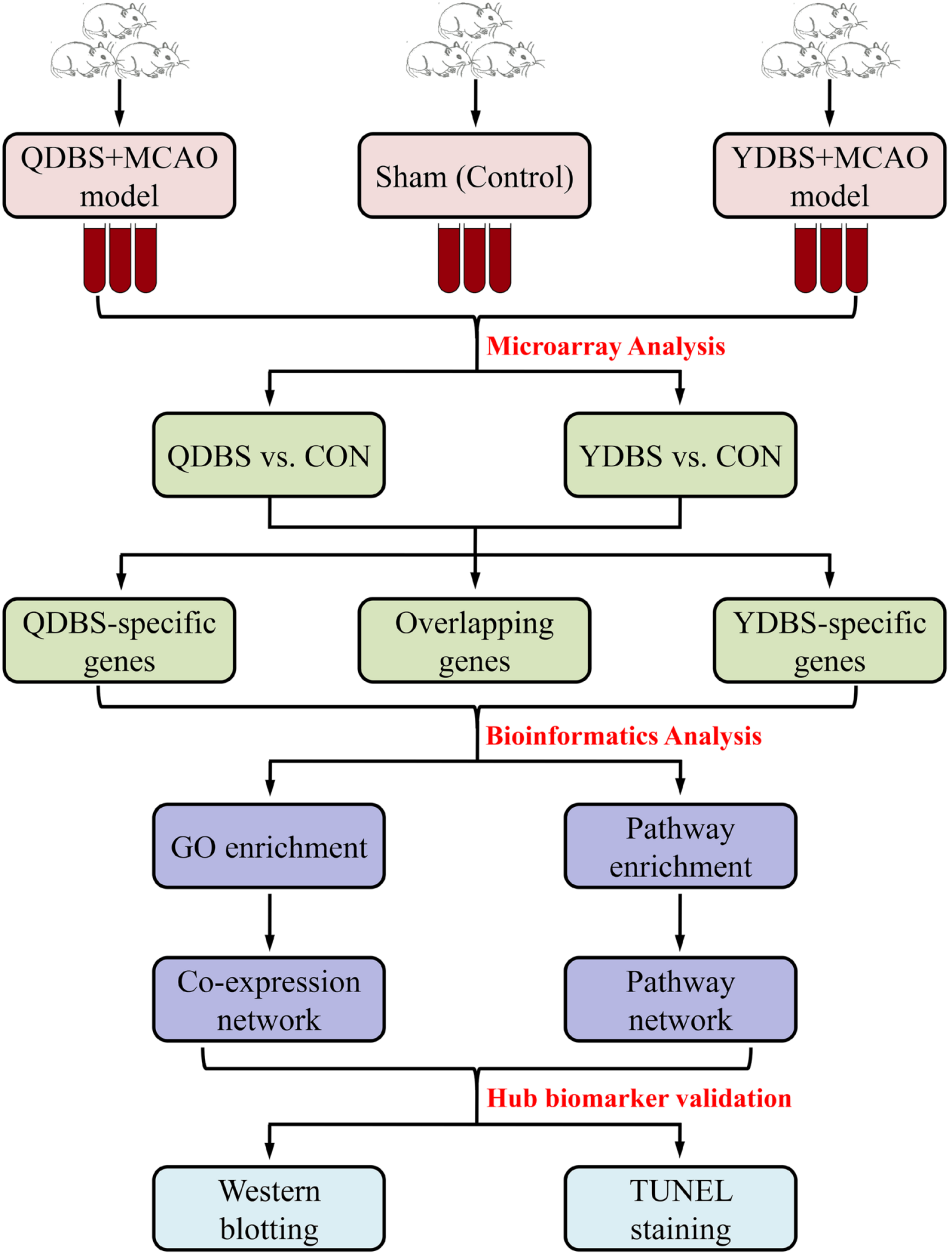
The flowchart of model establishment, microarray and bioinformatics analysis in this study

In the present study, we compared the circulating gene expression prolife of ischemic stroke rats with QDBS and YDBS based on transcriptomics. QDBS-specific genes, YDBS-specific genes and blood stasis common genes were obtained, the key genes and pathways of which were identified by the construction of networks. Finally, the hub biomarkers were further validated by investigating the protein expression level. The flowchart of this research was shown as Figure 1.

## RESULTS

### The morphological changes of ischemic stroke rats with QDBS or YDBS syndrome

**Figure 2:**
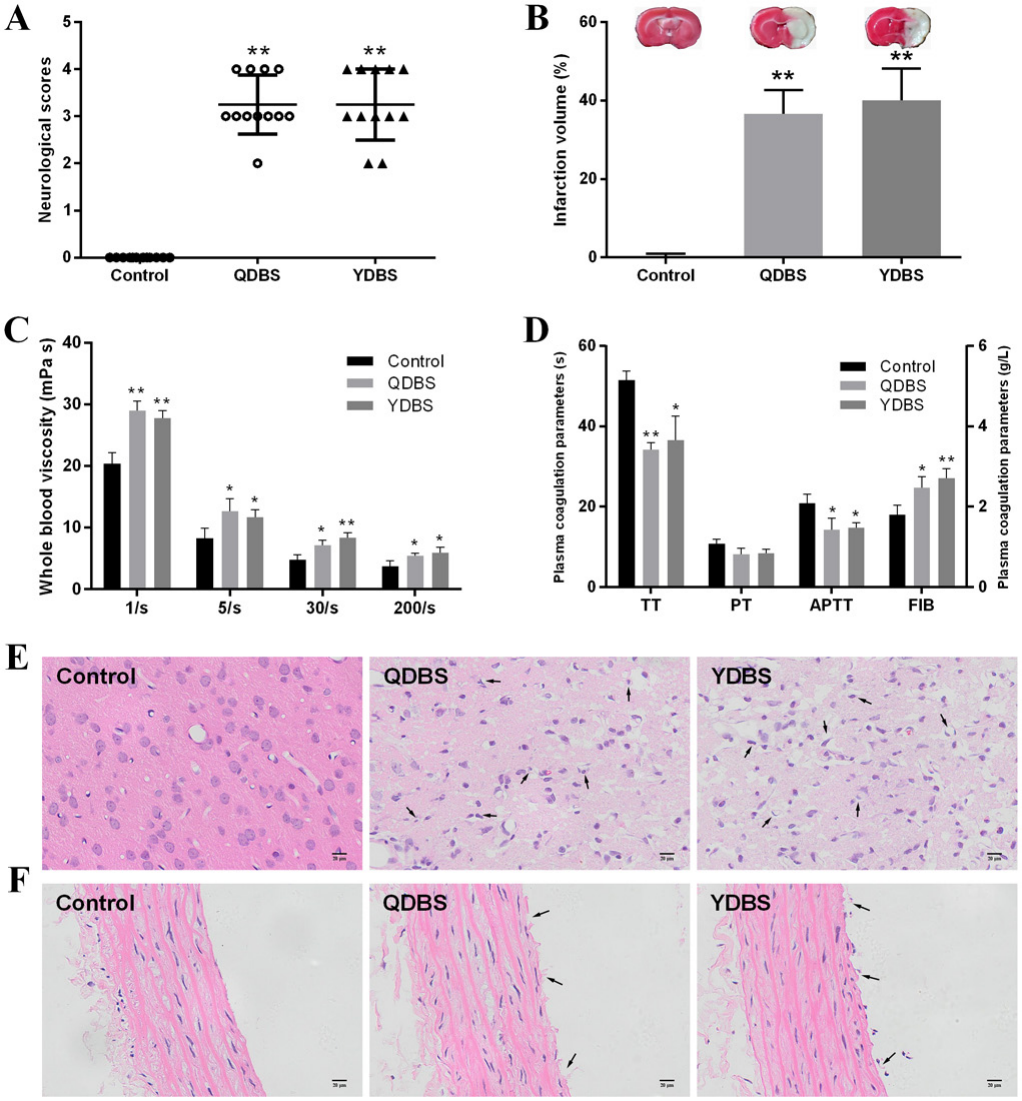
The morphological changes of ischemic stroke rats with QDBS or YDBS syndrome **(A)** The neurologic score of rats after 24 h reperfusion (n=12). **(B)** The brain infarct volume after the neurological tests (n=6). **(C)** Whole blood viscosity (WBV) of shear rates’ varying from 1 to 200/s (n=6). **(D)** Coagulation parametersof plasma (n=6). The left Y-axis represents PT, TT and APTT value. The right Y-axis represents FIB value. Data presented as mean ±SD. ^*^P < 0.05, ^**^P < 0.01 compared with control group. Histopathological observation of cerebral cortex **(E)** and thoracic aorta **(F)**.

Neurologic score and infarct volume are the two most important markers for brain injury. Compared with the control group, the rats’ neurologic score and brain infarct volume were increased in QDBS and YDBS model groups after 24 h reperfusion (Figure 2A and 2B).

It’s well accepted that hemorheological disorders are closely related to blood stasis [26], so the whole blood viscosity and coagulation parameters were detected. Results indicated that whole blood viscosity (WBV) significantly increased at all shear rates in the model groups (Figure 2C), which means the decrease of the blood flow velocity. Meanwhile, in the model groups, TT, PT and APTT were shortened while FIB content increased significantly when compared to the control group (Figure 2D).

Histopathological examinations were also performed to confirm if models were successful. The H&E staining showed that there were signs of disordered neurons arrangement, pyknotic and shrinkage of nucleus with widened pericellular spaces, presence of numerous vacuolated spaces and neuronal loss in model groups (Figure 2E). Consistent with these pathological changes in brain, the morphological alterations of thoracic aorta in QDBS and YDBS rats were also observed. The H&E stained micrographs revealed that the lumen of thoracic aorta in model groups were obstructed and microthrombus formed. Moreover, parts of vascular endothelial cells detached from the vascular walls (Figure 2F). No statistically significant differences were observed between the QDBS and YDBS groups.

### Identification of differentially expression genes of ischemic stroke rats with QDBS or YDBS syndrome

**Figure 3:**
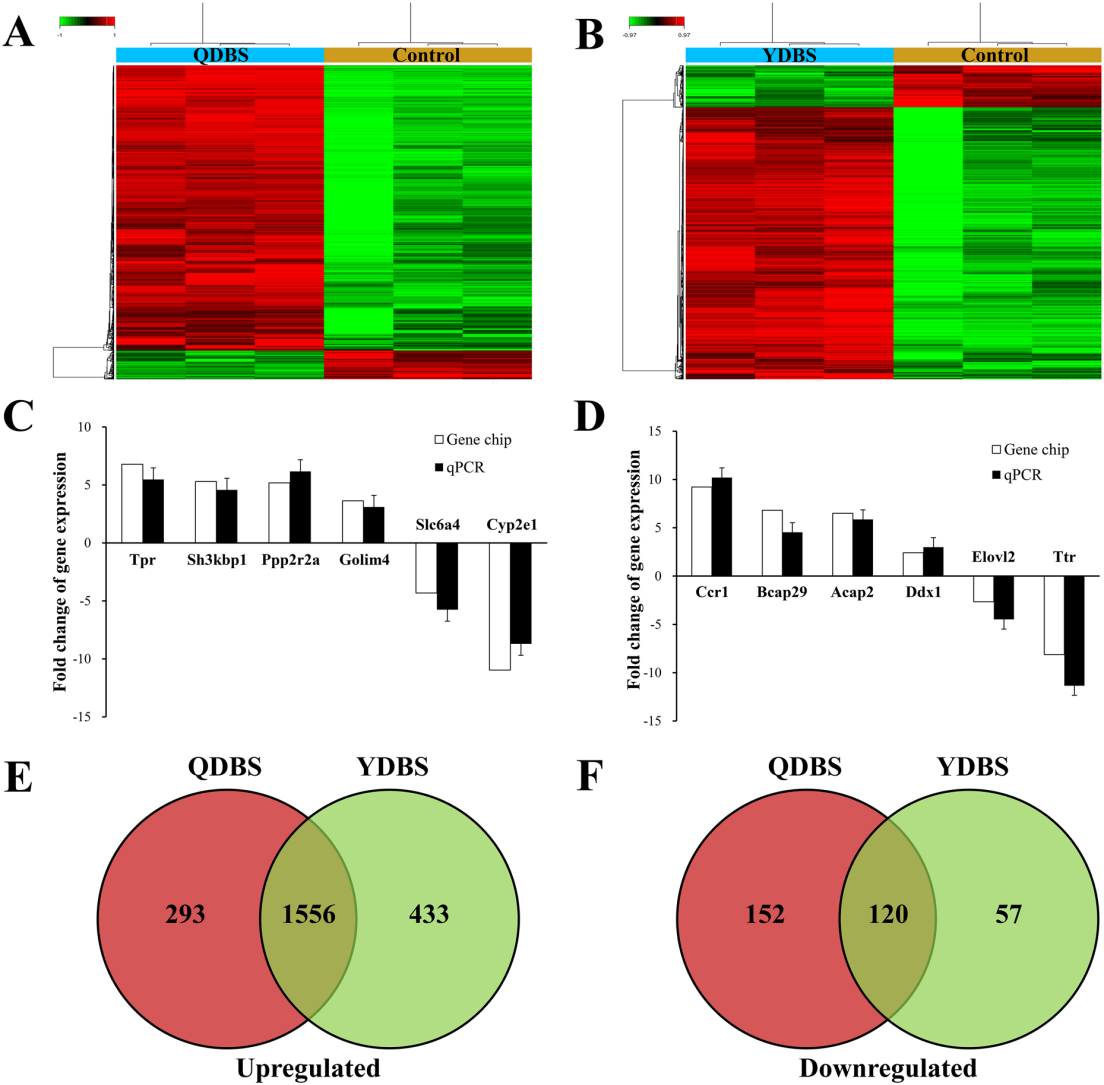
The differentially expressed genes (DEGs) among the groups Hierarchical clustering between QDBS **(A)** or YDBS **(B)** and control group. Green color represent down-regulated genes, red color represent up-regulated genes (P<0.05). Validation of a subset of genes differentially expressed between QDBS **(C)** or YDBS **(D)** and control group by qPCR. White bars represent the fold change at the expression level as indicated by microarray analysis; black bars represent the mean fold change of gene expression calculated by qPCR method. Values are the mean ± SEM (n = 6). The Venn diagram of up-regulated **(E)** and down-regulated **(F)** genes between QDBS/control and YDBS/control.

According to the cut-off criteria of |log_2_-fold change (FC)|> 1 and false discovery rate (FDR) <0.05, a total of 2121 differentially expression genes (DEGs) were detected in QDBS when compared to control group, 1849 genes of them were up-regulated and 272 were down-regulated (Figure 3A). 2166 genes were identified dysregulated in YDBS when compared to control group, including 1989 up-regulated genes and 177 down-regulated genes (Figure 3B). The qPCR results were all in accord with the microarray analysis (Figure 3C and 3D). Between the 2121 dysregulated genes in QDBS and 2166 dysregulated genes in YDBS, 445 were identified as QDBS-specific genes (293 genes were up-regulated and 152 were down-regulated), which indicated a special pathomechanism of ischemic stroke rats with QDBS syndrome. 490 were identified as YDBS-specific genes (433 genes were up-regulated and 57 were down-regulated), which maybe explain the pathomechanism of ischemic stroke rats with YDBS syndrome. Notably, 1676 genes (1556 genes were up-regulated and 120 were down-regulated) were overlapping between the dysregulated genes in QDBS and YDBS, which suggested a common mechanism underlying BSS (Figure 3E and 3F).

### Gene ontology and pathway analyses of the difference between QDBS and YDBS stroke rats

QDBS-specific genes, YDBS-specific genes and blood stasis common genes were uploaded to DAVID (Database for Annotation, Visualization and Integrated Discovery) software to perform gene ontology (GO) and KEGG (Kyoto Encyclopedia of Genes and Genomes) analyses. GO analysis revealed that 65 significant GO terms (FDR<0.05) were regulated by QDBS-specific genes, while 70 terms were regulated by YDBS-specific genes and 376 terms were regulated by the common genes. The top ten significantly enriched GO terms in the three subsets were presented in Figuer 4A, 4C, 4E. Among them, aging (*P*-value=3.70E-08), inflammatory response (*P*-value=2.93E-12) and DNA-dependent transcription (*P*-value=9.65E-33) were the most obvious.

**Figure 4:**
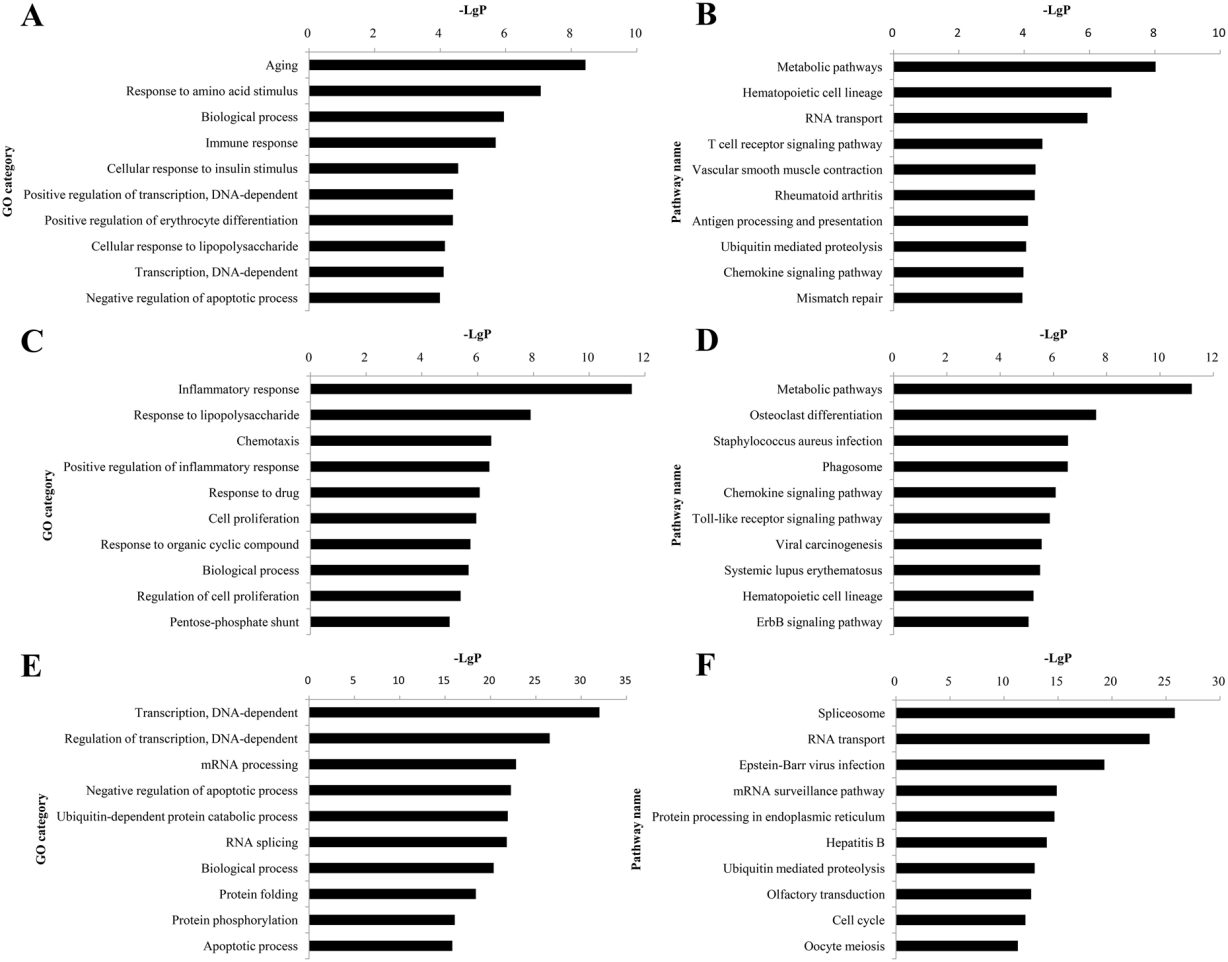
Histogram of GO and pathway enrichment analyses of dysregulated genes GO analysis of QDBS-specific genes **(A)**, YDBS-specific genes **(C)** and overlapping genes **(E)**. Pathway analysis of QDBS-specific genes **(B)**, YDBS-specific genes **(D)** and overlapping genes **(F)**. X axis, negative logarithm of the P-value (− LgP). Y axis, name of the GO or pathway items.

Pathway analysis demonstrated that there were 79, 87 and 138 pathway categories (FDR<0.05) were affected by the three subsets of genes. Figure 4B, 4D, 4F showed the top ten significantly pathways. The metabolic pathways (*P*-value=9.46E-09 in QDBS, *P*-value=6.46E-12 in YDBS) and Spliceosome pathway (*P*-value =1.51E-26 in overlapping) were enriched as the most significant ones according to the *P*-value.

### Pathway relation network analysis of the difference between QDBS and YDBS stroke rats

**Figure 5:**
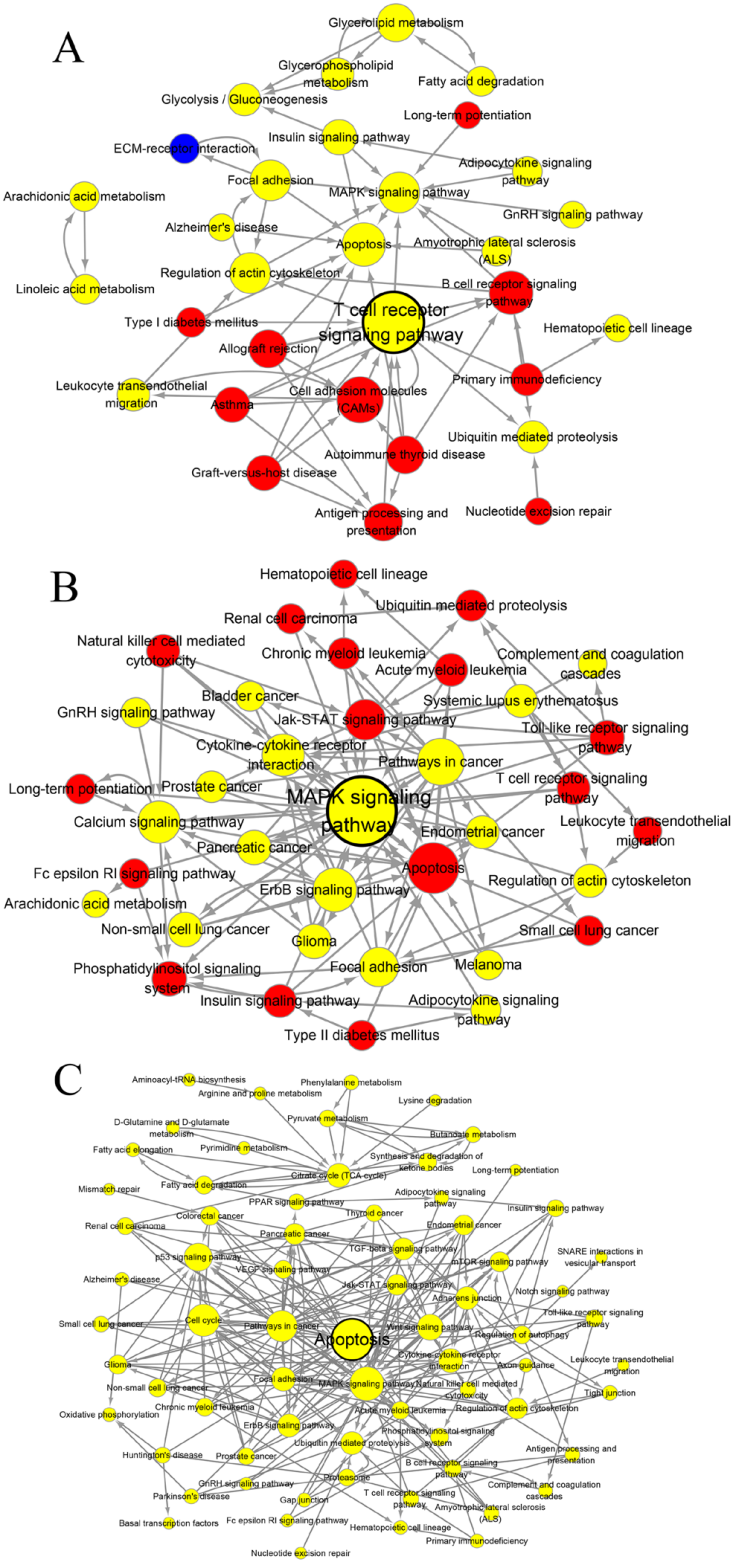
The interaction net of the significant pathways The pathway relation network of QDBS-specific genes **(A)**, YDBS-specific genes **(B)** and overlapping genes **(C)**. Nodes represent pathways. The area of nodes displays the degree that is the number of other genes that interact with this gene. Lines indicate interactions between pathways, where pathways indicated by the arrowhead are regulated by pathways of the arrow tail. Red nodes represent up-regulated pathways, blue nodes represent down-regulated pathways, and yellow nodes represent the up/down-regulated pathways. Nodes with black borders indicate the hub pathways identified by networks.

In order to define functional relationships among pathways enriched above, the pathway relation network analysis was conducted. As shown in Figure 5, the QDBS-specific pathway network was composed of 31 nodes (represent pathways) and 62 edges (line connections between pathways), while YDBS-specific pathway network contained 36 nodes and 112 edges. Besides, 72 nodes and 251 edges were assigned to BSS common pathway network. The T cell receptor signaling pathway, MAPK signaling pathway and apoptosis pathway were identified as the hub pathways, respectively.

### Gene co-expression network analysis of the difference between QDBS and YDBS stroke rats

**Figure 6:**
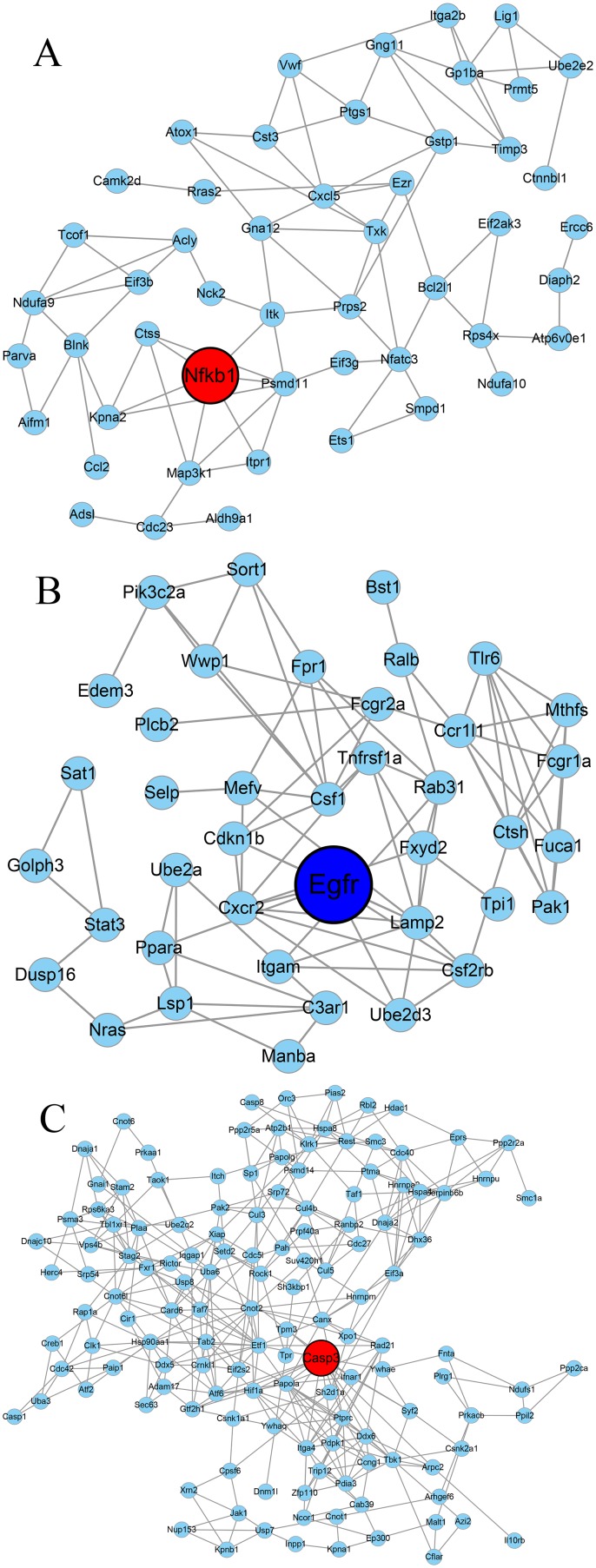
The co-expression network analysis of differentially expressed genes The co-expression network of QDBS-specific genes **(A)**, YDBS-specific genes **(B)** and overlapping genes **(C)**. Nodes denote genes; Lines represent gene-gene interrelation; the size of the nodes represents the degree value. Red nodes represent up-regulated genes, dark blue nodes represent down-regulated genes, and yellow nodes represent the up/down-regulated genes. Nodes with black borders indicate the hub genes identified by networks.

The gene co-expression network was constructed with respect to gene function associations (23), As shown in Figure 6, 50 nodes (represent the DEGs) and 84 edges (line connections between nodes) were assigned to the QDBS-specific co-expression network, and *Nfκb1* (FC=2.23, according to the identification of DEGs) was identified as the hub gene. YDBS-specific co-expression network contained 40 nodes and 88 edges with the hub gene *Egfr* (FC=-2.08). BSS common co-expression network was composed of 212 nodes and 474 edges, and *Casp3* (FC=2.82 in QDBS, FC=4.35 in YDBS) was the hub gene according to the degree size.

### Validation of the hub genes and pathways

**Figure 7:**
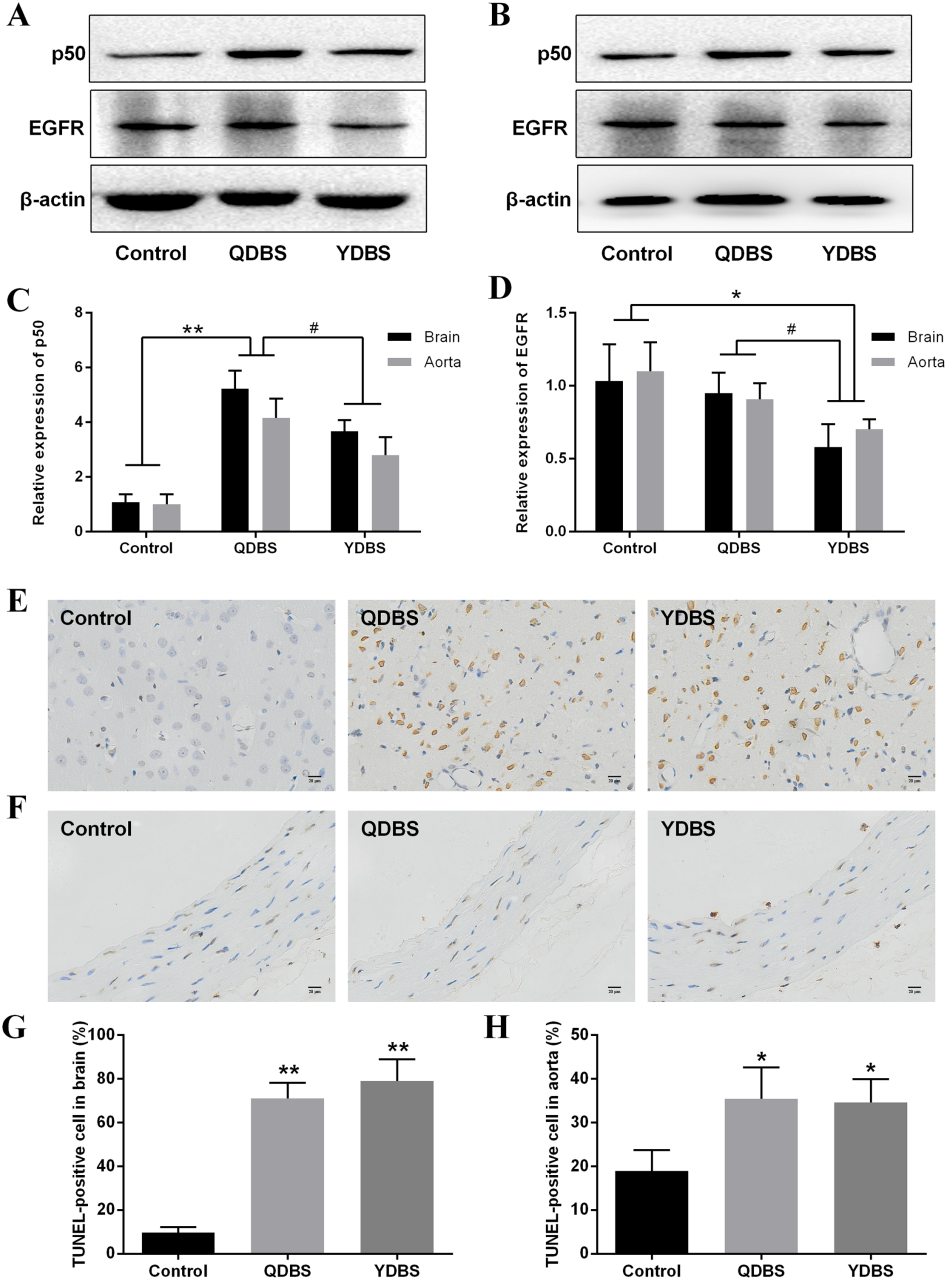
Validation of the hub genes and pathways Expression of NF-κB p50 and EGFR in brain tissue **(A)** and thoracic aorta **(B)** were detected by western blotting. The relative expression levels of p50 **(C)** and EGFR **(D)**. Tunel staining of cerebral cortex (E) and thoracic aorta **(F)**. Quantitative analyses of TUNEL-positive cells in brain tissue **(G)** and thoracic aorta **(H)**. Error bars: ± S.D (n=6). ^*^P < 0.05, ^**^P < 0.01 compared with control group. ^#^P < 0.05 compared with another model group.

The hub genes and pathways were validated both in brain tissue and thoracic aorta. Western blotting results showed that NF-κB1 (p50) expression level in QDBS group was significantly higher than control and YDBS group, while EGFR expression level was significantly decreased in YDBS group compared to control and QDBS group (Figure 7A-7D). The expression of p105 was too low to analysis from the protein band, so the statistical analysis of NF-κB1 was mainly based on the expression level of p50. In order to examine the apoptosis level of ischemic stroke rats with BSS, TUNEL staining was used to quantify apoptotic cells. The data in Figure 7E-7F showed that there were massive TUNEL positive staining in both brain tissue and thoracic aorta of the two model groups.

## DISCUSSION

Effectiveness of TCM in the treatment of ischemic stroke depends on the accuracy of syndrome differentiation [27]. However, as the two major symptoms of ischemic stroke, QDBS and YDBS symptoms are subjective and difficult to evaluate objectively. In our study, the existing diagnostic methods, including neurologic score, infarct volume, hemorheological parameters and histopathological examination, were failed to distinguish this two symptoms. Therefore, a standard diagnostic criterion with reliable biomarkers, circulating mRNA expression profiling, was performed to identify the dysregulated genes between ischemic stroke rats with QDBS and YDBS. By intersecting the DEGs between QDBS/control and YDBS/control, three subsets of dysregulated genes were obtained: 445 QDBS-specific genes, 490 YDBS-specific genes and 1676 blood stasis common genes, which indicated the diverse pathological mechanisms of ischemic stroke rats with different syndromes.

GO analyses were conducted for understanding the main function of DEGs. The results revealed that the functions of QDBS-specific genes were related to aging, response to amino acid stimulus, biological process and immune response. As for the YDBS, the functions of most differentially regulated genes were mainly related to inflammatory response, response to lipopolysaccharide, chemotaxis and positive regulation of inflammatory response. Our analysis also yielded 65 significant GO terms in commonly regulated genes, including DNA-dependent transcription, mRNA processing and negative regulation of apoptotic process, which indicated that the mechanism for ischemic stroke with BSS might relate to the dysfunction of the above biological process.

Similar to the GO analysis, pathway enrichment analyses were performed to further investigate the DEGs from another perspective. We found that metabolic pathways were both enriched to be the most significant pathway of QDBS and YDBS-specific genes. It’s probably because metabolic is the basis state of physiological and pathological in life, and the metabolic pathways is an integrated network contains a large amount of molecules based on the KEGG pathway. Thus, it’s reasonable to infer that the DEGs were easily enriched to metabolic pathways. However, the GO and pathway enrichment are preliminary analyses of DEGs, whether they play key roles in the progression of ischemic stroke is still need to be verified by network analysis.

According to the pathway and gene co-expression network of QDBS-specific genes, T cell receptor signaling pathway and *Nfκb1* were at the core of networks. After stroke, myelinreactive antigens leak out of the brain with the breakdown of the blood–brain barrier, which induces rapid activation of the immune system [28]. It causes the recruitment of monocytes, neutrophils and T cells into the brain [29]. Among these, T cells are found in the brain within hours after stroke, which may play a significant role in exacerbating ischemic injury [30]. The transcription factor NF-κB is a key regulator of hundreds of genes involved in inflammation [31]. It consists of five different subunits, including p65 (RelA), RelB, c-Rel, p50/105 (NF-κB1), and p52/p100 (NF-κB2), form homo- and heterodimers in various combinations. There is ample evidence that NF-κB is activated in cerebral ischemia [32]. Nuclear translocation of p50 and p65 were observed in the brain areas surrounding the necrotic infarct core of patients who suffered a stroke or rats subjected to middle cerebral artery occlusion (MCAO) [31, 33], while the inhibition of NF-κB in neurons resulted in a significant and comparable reduction in infarct size in both transient and permanent stroke models [34, 35], suggesting that NF-κB plays a detrimental role in cerebral ischemia. Meanwhile, inflammatory response and positive regulation of NF-κB transcription factor activity were found to be closely related to QDBS [36]. Our results showed that *Nfκb1* was significantly up-regulated in ischemic stroke rats with QDBS rather than YDBS. Thus, *Nfκb1* could be considered to be a discrimination factor and therapeutic target for QDBS.

In terms of YDBS-specific genes, MAPK signaling pathway and *Egfr* were identified as the hub pathway and gene based on the network analyses. The results are in good agreement with another integrated analysis of expression profile in MCAO animal models, in which MAPK was also enriched as the most significantly pathway [37]. MAPK has been recognized as a potential therapeutic target for ischemic stroke, activation of MAPK signaling after ischemic stroke has been identified in neuron, astrocyte and microglia [38], and the inhibition of that has protective effect on ischemic stroke [39]. Besides, it has been suggested that MAPK signaling pathway played an important role in the ischemic stroke induced astrogliosis [40]. As an important signal molecule of MAPK signaling pathway, the epidermal growth factor receptor (EGFR) subfamily consists of four closely related tyrosine kinase receptors, including ErbB1, ErbB2, ErbB3, and ErbB4. On ligand binding, the receptors form functional dimmers and activate various signaling modules and their downstream targets, and ultimately regulating cell proliferation, differentiation, migration, and matrix homeostasis [41]. EGFRs have pleiotrophic actions on central nervous system cells, researches showed that reduced EGFR signaling decreased remyelination and oligodendrogenesis [42]. In our study, EGFR expression level was found to decrease from 24h postischemia in the ipsilateral brain side, which is consistent with previous report [43]. This may be due to a large amount of neuronal loss in the infarction zone. In addition, EGFR could be used as a blood-based diagnostic biomarker for ischemic stroke [44].

Apoptosis contribute to a significant proportion of neuron death following ischemic stroke [45], so it’s not surprising that apoptosis was identified as a hub pathway in blood stasis common genes of ischemic stroke. During ischemic stroke, overproduction of free radicals and excessive influx of Ca2^+^ activate caspase-3, a cysteine protease that plays an effector role in apoptosis, eventually resulting in neuronal dysfunction and cell death by necrosis or apoptosis [46–48]. Previous research suggested that plasma caspase-3 levels were higher in stroke patients compared with control group, and also positively correlated with neurological score and infarct growth [49]. Following transient focal cerebral ischaemia, mice overexpressing human caspase-3 exhibited increased apoptosis and larger lesion volumes than wild-type animals [50], whereas mice with genetic deletion of caspase-3 showed smaller infarct volumes in response to brain ischaemia [51]. Furthermore, a panel of plasma biomarkers was evaluated and caspase-3 appeared to be the most promising to achieve a rapid biochemical diagnosis biomarker of stroke [52]. According to a network analysis, caspase-3 as well as the corresponding apoptosis pathway were identified to be one of the therapeutic targets related to blood stasis [12].

Finally, the hub genes and pathways obtained from networks above were further validated by western blotting and TUNEL staining analysis. Since the pathological change of aorta is a crucial indicator for blood stasis [53, 54], both the brain tissue and thoracic aorta were tested in our study. As expected, the results were coincident with the microarray findings, suggesting that these hub modules might be potential biomarkers of ischemic stroke rats with BSS.

Undeniably, there were several limitations of the present study that should be considered. The main limitation was the relatively small sample size. Although there were twelve animals in each group, the samples for microarray analysis were only three, which might increase the variation within groups. In addition, subjects in the study were rats instead of patients, the potential biomarkers obtained from rats may not fully apply to human beings. Of course, further studies with larger-scaled samples of clinical patients are needed to confirm our findings.

In this study, we investigated the difference between ischemic stroke rats with QDBS and YDBS syndrome by transcriptomics and bioinformatics analysis. Three subsets of dysregulated genes, including QDBS-specific genes, YDBS-specific genes and blood stasis common genes were obtained. *Nfκb1*, *Egfr* and *Casp3* were identified as the hub genes, while T cell receptor, MAPK and apoptosis pathway were the hub pathways, respectively. We achieved a better understanding on the biological characteristics of ischemic stroke with QDBS and YDBS syndrome, and transcriptomics presented itself to be a promising tool for the syndrome differentiation. The proposed biomarkers might provide insight into the accurate diagnose and proper treatment for ischemic stroke with BSS.

## MATERIALS AND METHODS

### Ethics statement

Investigation has been conducted in accordance with the ethical standards and according to the Declaration of Helsinki and according to national and international guidelines and has been approved by the Fourth Military Medical University Committee on Animal Care.

### Animals and models

Male Sprague-Dawley (SD) rats, weighing 250–280g, were supplied by Medical Laboratory Animal Center, Fourth Military Medical University, Xi’an, China. Rats were kept in plastic cages at 22 ± 2°C with free access to pellet food and water and on a 12 h light/dark cycle. After 7 days of acclimatization, the animals were randomly divided into three groups (control group, QDBS group, and YDBS group) with twelve animals in each.

The BSS models were produced as described previously [55]. Briefly, rats in QDBS group were treated with exhaustive swimming exercising once a day for 21 days so that they were in a chronic state with QDBS [56]. While the YDBS model rats were intramuscularly injected with hydrocortisone (45mg/kg) one time per day for 13 days, followed by subcutaneous injection of epinephrine (0.36mg/kg) for 1 day [57, 58]. On the next day of that, the middle cerebral artery occlusion (MCAO) was performed as described previously [25]. In brief, rats were anesthetized with 5% isoflurane in 30% oxygen / 70% nitrous oxide, and anesthetization was maintained with 0.5% isoflurane during surgery. The right common carotid artery and the external carotid artery (ECA) were exposed. The right internal carotid artery (ICA) was dissected. Then a 4-0 monofilament nylon suture (Doccol, Sharon, MA, USA) was introduced from ECA to ICA to occlude the right middle cerebral artery. The nylon thread was slowly withdrawn 2 h after the induction of ischemia. Control group rats went the same surgical procedures except the monofilament insertion.

### Neurological examination and infarct assessment

At 24h after reperfusion, a 0–5-point scale neurological reported previously [59, 60] was used for neurological evaluation by an investigator who was blind to the experimental condition of the animals. 0=no neurological dysfunction; 1=failure to extend left forelimb fully when lifted by tail; 2=circling to the contralateral side; 3=falling to the left; 4=no spontaneous walk or in a comatose state; 5=death.

Six rats of each group were deeply anesthetized after the neurological tests. After the blood was harvested, the brain was then removed to assess the infarct volume. The brain was cut into 2-mm sections. Sections were immersed in 2% 2,3,5-triphenyltetrazolium chloride (TTC) (Sigma-Aldrich, St. Louis, MO, USA) at 37°C for 20 min and then placed in 10% formaldehyde. Total infarct volume was calculated by a blinded investigator using an image analysis system (Adobe Photoshop 9.0, Adobe Systems Incorporated, San Jose, CA, USA). Edema correction of infarct volume was calculated using the following equation [61]: Vedi=Vinfarct× (1−(Vipsi−Vcontra)/Vcontra); Vedi, volume edema corrected infarct; Vinfarct, volume infarct; Vipsi, volume ipsilateral hemisphere; Vcontra, volume contralateral hemisphere.

### Whole blood viscosity and coagulation parameters determination

The rest of rats in each group were anesthetized after the neurological tests, and blood was drawn from the abdominal aorta. About 2ml blood was collected in plastic tube with 3.8% sodium citrate (citrate/blood: 1/9, v/v) for RNA extraction. 2ml blood was collected into dry vacuum tubes with heparin lithium for WBV. 2ml blood in plastic tube with 3.8% sodium citrate for coagulation parameters. Blood was centrifuged at 3000 rpm for 15 min to obtain plasma. FIB, PT, TT and APTT were measured by the automatic instrument of coagulation (ACL TOP700, Instrumentation Laboratory, USA). The WBV was determined with a cone-plate viscometer (Model ZL9000, Zonci, Co., China) at different shear rates maintained at 37°C. WBV was measured with shear rates’ varying from 1 to 200/s. All experiments were completed within 3h after blood collection.

### Histopathological observation

Rats were deeply anesthetized and then perfused with 4°C physiological saline solution, followed by 4% (v/v) paraformaldehyde. The brain and thoracic aorta were removed and fixed with 4% paraformaldehyde for 24 h. Then the tissue were embedded in paraffin and cut into sections of 5 μm thickness. The sections were stained with hematoxylin and eosin (H&E) for histopathological observation. Apoptosis level was determined by a terminal deoxynucleotidyl transferase-mediated dUTP nick-end labeling (TUNEL) detection kit (Beyotime Institute of Biotechnology Co., Ltd Haimen, China) according to the manufacture’s protocol.

### Western blotting analysis

Brain tissues and thoracic aortas were harvested after the rats were sacrificed. The total proteins were extracted and separated via sodium dodecyl sulfate polyacrylamide gel electrophoresis (SDS-PAGE). Transferred onto nitrocellulose membranes and probed with the antibodies against: NF-κB p105/50 (1:1000, Abcam, Cambridge, MA, USA), EGFR (1:1000, Abcam, Cambridge, MA, USA). β-actin was taken as the loading control. The relative optical densities of the specific proteins were determined with Quantity One software (Bio-Rad Laboratories Inc., Hercules, CA, USA) and quantified using ImageJ software (NIH, USA).

### RNA extraction and microarray analysis

Total RNA in the blood was isolated using TRIpure LS blood (liquid sample) RNA extraction reagent (BioTeke, China) according to the manufacture’s protocol. The RNA content was examined by identifying A260 and A280 values by using an Nanodrop 2000 (Thremo Scientific, Waltham, MA, USA), and the RNA integrity was assessed by using a 2100 Bioanalyzer (Agilent Technologies) and an RNA 6000 Nano Kit (Agilent Technologies). Total RNA was processed for double-strand cDNA synthesis, IVT and cRNA fragmentation with GeneChip 3’IVT Express Kit (Affymetrix, Santa Clara, CA, USA) according to the manufacturer’s instruction. Then, it was processed for hybridization with the GeneChip^®^ Rat Gene 2.0 ST Array (Affymetrix) by using a GeneChip^®^ Hybridization, Wash and Stain Kit. Finally, the arrays were washed and scanned by an Affymetrix GeneChip Command Console. These microarray data have been deposited in NCBI Gene Expression Omnibus (GEO) under accession number GSE100235.

### Data preprocessing and differential analysis

The raw files were transformed to probe-level data, a Robust Multi-chip Average method was used to compute expression levels of probes. The t-test method was used to calculate the *P*-values of genes, which were later adjusted to false discovery rate (FDR) according to Benjamini-Hochberg’s method [62]. The screening standards of distinctly significant gene were |log_2_FC|> 1 and FDR<0.05 [63, 64]. Hierarchical clustering analyses were performed according to the literature methods [65]. The DEGs between QDBS/control and YDBS/control were intersected, and three sets of dysregulated genes were achieved: QDBS-specific genes, YDBS-specific genes and blood stasis common genes.

### Quantitative RT-PCR validation

The RNA extraction and RNA integration inspection procedures were mentioned above. Total RNA were processed for cDNA synthesis using M-MLV reverse transcriptase (Promega Corporation, Madison, WI, USA). cDNA was amplified by PCR in Stratagene MX3000p (Agilent Technologies, Santa Clara, CA, USA) using SYBR Master Mixture (TaKaRa, Tokyo, Japan). The expression levels of target genes were determined against the levels of GAPDH and calculated using the 2-∆∆Ct method. All the assays were performed in triplicate. The sequences of the primers used in PCRs are shown in Supplementary Table 1.

### Gene ontology and pathway enrichment analyses

GO analysis could predict the gene function in molecular function, biological processes and cellular components, which has been applied for annotating genes of high-throughput genomic or transcriptomic data [66]. KEGG is a recognized pathway-related database for systematic analysis of gene functions [67]. In the present study, the DAVID database was applied to implement GO and KEGG pathway enrichment analyses. *P* <0.05 and FDR<0.05 were considered to be statistically significant.

### Network construction and hub module identification

Pathway relation network could identify the key pathway that regulates the upstream and downstream pathways simultaneously. Gene co-expression network could help to seek the key gene of regulation and interaction venation thoroughly. In networks, hub node has more complex correlativity compared with others, which suggesting that it may play significant role in the underlying mechanisms of disease [68]. The networks were carried out by Genminix Informatics (Shanghai, China) [69], and the algorithms mainly referenced published methods [70–72].

### Statistics analysis

Statistical analyses were carried out with SPSS 19.0 (SPSS Inc., Chicago, IL, USA). Assays were conducted at least three times unless otherwise stated. All values were expressed as either a mean ± standard deviation (SD) or mean ± the standard error (SE), except for the neurobehavioral score. The comparison of data between groups were analyzed using a one-way analysis of variance (ANOVA) followed by Tukey’s multiple-comparison test. Neurobehavioral scores were expressed as the median (interquartile range, IQR) and were analyzed using the Mann–Whitney U test. *P* < 0.05 was considered as statistical significance.

## SUPPLEMENTARY MATERIALS TABLE



## Abbreviations

BSS: blood stasis syndrome
DAVID: database for annotation, visualization and integrated discovery
DEGs: differentially expressed genes
FC: fold change
FDR: false discovery rate
GO: gene ontology
KEGG: Kyoto encyclopedia of genes and genomes
MCAO: middle cerebral artery occlusion
QDBS: Qi deficiency blood stasis
TCM: traditional Chinese medicine
TUNEL: terminal deoxynucleotidyl transferase-mediated dUTP nick-end labeling
WBV: whole blood viscosity
YDBS: Yin deficiency blood stasis
