# Supplementary material for: Differential gene expression profiles between two subtypes of ischemic stroke with blood stasis syndromes
Source: Oncotarget. 2017 Dec 4;8(67):111608–22. doi: 10.18632/oncotarget.22877 (PMC5762346; doi:10.18632/oncotarget.22877)
Supplement: Supplementary file 1 [file oncotarget-08-111608-s001.pdf]

Differential gene expression profiles between two subtypes of ischemic stroke with blood stasis syndromes

SUPPLEMENTARY MATERIALS

Supplementary Table 1 : Primers used in real-time RT-PCR

Gene	Forward primer (5'-3')	Reverse primer (5'-3')
Tpr	ctcaagaccgcaatctaggc	cagcttctaactcttcctttgctc
Sh3kbp1	ctctgctttggcgactcag	tgggcctggtaatcaaactc
Ppp2r2a	ggtgggagagtcgtcatctt	tctcctctgctatgagactgga
Golim4	gtgatggcagggaatcca	tttttctcttcagtcaaatcttcct
Slc6a4	atcacctggacgctgcat	tggatctgcaggacatgg
Cyp2e1	cgcatggaattgtttctgc	tccttagggtcaaccagagact
Ccr1	tggattgactacaagctgaaaga	aaacccagagagaagcttacaca
Bcap29	gcagacctgctgcctatga	cgtaaccagacgtctcaacact
Acap2	agggctgctttggaagaagt	tcaatcatcgcgatacaaagtt
Ddx1	tgtgacaacttggagcagtacttt	cacatgagaattggtgtcctttt
Elovl2	gctacaacttgcagtgtcagaatc	accacaagaccttggctacc
Ttr	gcttcccttcgcctgttc	gacacttggattctccagcac
Gapdh	atgggaagctggtcatcaac	catttgatgttagcgggatct
